# Polarization in social media assists influencers to become more influential: analysis and two inoculation strategies

**DOI:** 10.1038/s41598-019-55178-8

**Published:** 2019-12-09

**Authors:** Ivan Garibay, Alexander V. Mantzaris, Amirarsalan Rajabi, Cameron E. Taylor

**Affiliations:** 1 University of Central Florida, Department of Industrial Engineering and Management Systems, Orlando, 32816 USA; 2 University of Central Florida, Department of Statistics and Data Science, Orlando, 32816 USA; 3 University of Central Florida, Department of Computer Science, Orlando, 32816 USA

**Keywords:** Mathematics and computing, Statistics

## Abstract

This work explores simulations of polarized discussions from a general and theoretical premise. Specifically the question of whether a plausible avenue exists for a subgroup in an online social network to find a disagreement beneficial and what that benefit could be. A methodological framework is proposed which represents key factors that drives social media engagement including the iterative accumulation of influence and the dynamics for the asymmetric treatment of messages during a disagreement. It is shown that prior to a polarization event a trend towards a more uniform distribution of relative influence is achieved which is then reversed by the polarization event. The reasons for this reversal are discussed and how it has a plausible analogue in real world systems. A pair of inoculation strategies are proposed which aim at returning the trend towards uniform influence across users while refraining from violating user privacy (by remaining topic agnostic) and from user removal operations.

## Introduction

The topic of polarization in societies is a problem of major concern and this problem has a different platform upon which it can develop with the availability of online social networks. Key differentiators are the low cost of communication with large groups of people over long distances and another often overlooked feature is the historical track record of popular content which can be on public display^1,2^. This last point is very important for the users who dedicate a substantial amount of their time as they are indicators for the rank of influence (‘views’, ‘re-tweets’, ‘likes’, etc). The effort put into obtaining influence can be assisted through *cooperation* from the content recipients when they repropagate messages(resharing or ‘re-tweet’). This can be expected to produce non-trivial complex aggregate behaviors^3,4^ and this work investigates how polarization (disagreements) can benefit users who are in pursuit of increasing their relative influence. Based upon the modeling approach which produces the effects where polarization can benefit a particular group of users, inoculation strategies are explored which reverse the induced effects to pre-polarization states.

The topics for polarization can come from a wide array of different areas of society such as political discourse^5,6^, where increases are recently being observed in the US^7–9^. Other sources of polarization can arise from topics such as wealth inequality^10–12^ that has been a challenge for a great deal of history but can now be studied as a topic of online engagement^13^. An exploration of the anatomy of the discussion surrounding the topics can be conducted with various modern tools such as latent Dirichlet allocation^14^ where key factors can be identified between categories of content and their ideological labels. There are various approaches to summarizing text from different groups so that the key differentiating features can be examined^15^.

It is uncertain as to whether that insight does provide any direction as to how a polarized discussion can be reduced, or even what is to be ‘reduced’ in the first place. There is literature on the approaches that can be taken to reduce polarization in social networks^16–20^, but there are challenges that commonly appear. These are that the approaches require human interpretation as to what directions to take by understanding the audience motivation^21^, that the content is visible to inspection^22^, or that particular users can be removed from the network participation^22^. The paper of ^23^ discusses how public group discussion can provide a means for reducing polarization but that this may not take place when it is done by representatives of opposing fractions. The author also discusses the incentives that representatives have and how the public discourse can be used for their private motives by influencing the opinions of others.

Our approach to addressing incentives does not assume that the contents of a message can be examined in order to adhere to privacy advocacy^24^ or depend upon a label on its state. It also explores methods in which polarization can be reduced, and reversed without the need to remove nodes. There are various reasons that this approach is sought and a key one is that no particular side of the argument is seen as a target by ‘overreaching’ authorities. It is assumed that these incentives for benefiting from polarization can be explored by the social media users even if the only feature they notice is their influence score changes over time. Although online discussion platforms differ in their mechanisms for sharing content a stochastic message exchange equation is proposed in which most platforms will share some aspect of that basic dynamic. Message exchanges in online social networking platforms where users are exposed to a pool of content and their sharing of content and can be considered equivalent to the popular retweet mechanism, is in effect an amplification of content by the original author in which various authorship credit features exist^25^.

This is a key aspect that can appeal to users, in that their messages do not need to be based upon platforms which have an association with large direct channels with high barriers of entry which convential content dissemination channels do (eg TV, newspapers or publishing houses). The work in^26^ describes this low investment to high influence return that can be sought on many modern platforms. The potential reach for independent profiles on these networks to have their content (and authorship acknowledgement) propagated through to millions if not possibly more, from a mix of directed edges and indirect propagations (cascade effect) is lucrative to say the least.

This has been seen in practice from a commercial perspective during the 2013 superbowl in which there was a power failure resulting in attendees using their phones for lighting in the stadium with social media apps engaged. Prior to the event, the Twitter Pepsi account was trending with the largest influence that then was reduced over time from the blackout. The accounts of Audi and notably Oreo cookies, managed to provide realtime content authoring with references to the power outage. This resulted in their accounts dominating the subsequent discussion^27^. Opportunities like this provide a lucrative investment from the perspectives of marketing strategists in finding low cost opportunities where large impacts on the viewership can be made.

The methodology section 4 describes the development for the framework upon which the model of social network members communicate and exchange messages. From the various models of communication proposed, the work of *Dynamic Communicators*^28^ is chosen to be extended as it offers an intuitively reasonable explanatory mechanism for how users exploit and utilize the ad-hoc sharing capabilities in online social platforms to derive more influence than their direct broadcast capabilities provide. It quantifies the effect of content sharing which produces ‘temporal walks’ created through message propagations over a time ordered network snapshots. The dynamic nature of temporal walks is explored in^25^ and^29^ is also applied to large commercial contexts of targeted advertising^30^. These studies support that effective users in social networks do not necessarily rely exclusively upon large bandwiths (many direct messages) to apply their influence upon other nodes but rely upon intermediaries to propagate their content between temporal adjacencies. Namely the effect where users producing low numbers of messages which then spread to a large number of users indirectly through intermediaries/relays. A key feature of the development proposed, is that the underlying influence^31^ parameter must change as a result of successes or failures when content is successfully disseminated through intermediaries. It is considered that over time the successes should produce a carry-on effect that is visible as a relative measure between other members in the network of influence success.

Given a model which represents a framework where users can exploit the resharing content mechanism/dynamics, within that framework is built the dynamics for how the users can hypothetically behave during a disagreement that creates the effect coined ‘polarization’ and is presented in Eq. 3. A key feature of the experiments performed in the Results section, is what changes affect community communication experience and how that flow of communication is altered. It is assumed that a more ‘level playing field’ is a positive sign of a healthy social gathering on any platform. Our results show that prior to polarization, the networks display a trend towards an increase in homogeneity in regards to the ability to disseminate content through proxy receivers. This can be interpreted as a result of a type of increased familiarity between users over time points and a more uniform evaluation of the content value.

The polarization phase, whose dynamics are represented by equations^3,4^, reverses this trend showing a favor in the content originating from those network members which had an initial high ranking influence scores. This allows a recapturing of the control of the dialogue via a feature of the dynamics that is akin to ‘intimidation’ of message exchanges during a polarization event. The Subsections 4.1 and 4.2 outline the inoculation strategies that will alleviate this effect as shown in the Results section. The proposed inoculation strategies do not assume content access, or do they require removal of accounts which are considered catalysts of the polarized content and have incentives from the polarized phase. The dynamics which produce a polarization are in agreement with the work of^32^ that shows how the formation of different opposing opinions can be observed in the simulations and in real world events.

The key aspect of the methodology which allows an examination of the ad-hoc content spread, is that the users all share the same rate at which content is produced and the number of people it reaches but the probabilities for the further propagation mediated by these contact events is regulated by a parameter of ‘influence’ (shown in Eq. 1). This parameter is allowed to increase with the number of successful responses (an indicator similar to retweet counts) and is introduce as described in Eq. 2. During the simulation the dynamics are modified in order to simulate a polarization event equations^3,4^ and is taken as a different phase along a simulation trace representing time. The effects from polarization produced are mitigated and reversed by introducing either one of 2 different inoculation strategies described in Subsections 4.1 and 4.2 that are content agnostic and privacy preserving.

## Results

The results of the simulations run using the methodology proposed in Section 4 are presented here, where the model is used to examine the effects and possible remedies for communication in social networks which has become polarized. A key feature of the simulations is the changes in the relative number of successful responses one group of nodes has in respect to another group as a result of a polarization phase being introduced. The distribution of the influence parameter values allocated from a cubic distribution along the nodes numbers ${l}_{n}:0 < {l}_{1}\le {l}_{2}\le \ldots \le {l}_{N}$. Nodes are assumed to produce content at an equal rate which does not go against the influence assignments as most platforms do not constrain or regulate user content dissemination based upon influence rankings.

**Figure 1:**
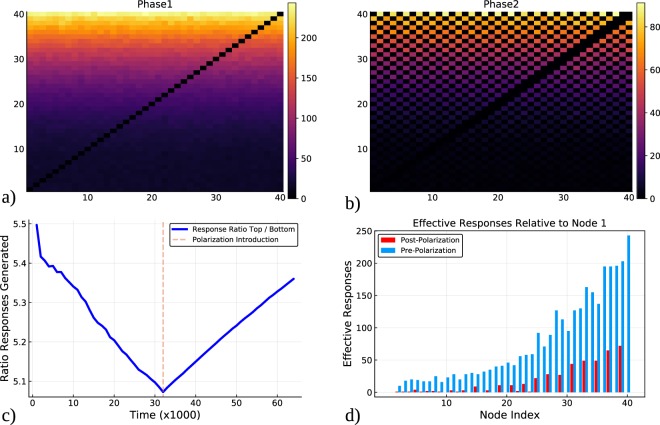
How influence scores change before and after polarization. Plot (a) shows the accumulated counts of the successful responses between nodes sending messages without polarization, and plot (b) shows the same information during the phase of polarized communication (evens and odds have a disagreement). Each cell represents the number successful messages sent by user *i* to user *j*. Plot (c) shows the value of the relative influence of the top half of the nodes in respect to the bottom half during the simulation in which a polarization event is introduced at the dashed vertical line. Plot (d) shows the total amount of successful messages sent by each user to the lowest ranked influencer (before and after the introduction of polarization).

**Figure 2:**
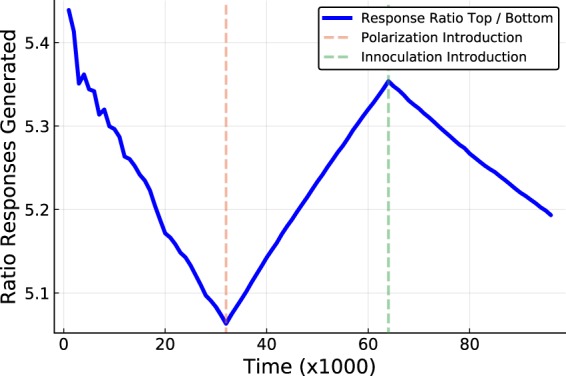
How *inoculation strategy 1* can reduce the polarization effect. The plot shows the changes in the values of the perceived influence that nodes use when deciding whether to propagate a message sent by another node in 3 phases of a discussion; the non-polarized discussion, the polarized discussion and the polarized discussion with the *inoculation strategy 1 applied*. A polarization event is introduced at the first dashed vertical line, and at the second vertical dashed line the *inoculation strategy 1* is applied.

**Figure 3:**
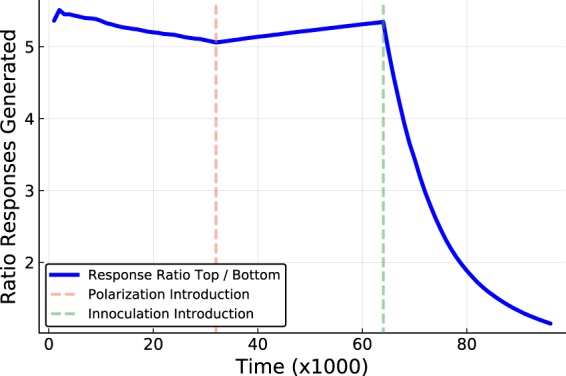
How *inoculation strategy 2* can reduce the polarization effect. The plot shows the changes in the values of the perceived influence of nodes during 3 phases of a discussion; non-polarized exchange, polarized exchange and the polarized exchange having *inoculation strategy 2* applied. This decrease in the thrid phase shows the capability for this inoculation strategy to return the network to a discussion with a more uniform distribution of dissemination capabilities across the participants.

The accumulation of influence is based upon the number of times another node has propagated a node’s content governed by a stochastic message passing equation. Figure 1 demonstrates a simulation with 2 phases of communication; without polarization and then with polarization. The effects upon aggregate groups is examined as well as for the individual nodes in the network of 40 synthetic users (discussed below). Figure 2 shows the results of a simulation with 3 phases of a simulated exchange; non-polarized, polarized and the continued polarized dynamics overlaid with that of *inoculation strategy 1*. Figure 3 also shows 3 phases of the simulated exchange; non-polarized, polarized and the continued polarized dynamics this time overlaid with that of *inoculation strategy 2*. The key feature of the inoculation strategy is that the disparity between users in having their content propagated through intermediates is reduced. The Supplemental Material discusses how the trace data was used to compute the measurements presented in the plots.

The number of nodes chosen is 40 according to the Dunbar number of community sizes where we can expect such a group to be able to message all other members directly and uniformly^33,34^. This number is also chosen in some of the related research into public discourse on political discussions such as^35^ which studies 40 blogger accounts that were deemed to be *A-list* in 2004 prior to the US presidential election. A separate blog analysis of opposing political blogs in^36^ also used 40 blogs which were all interlinked. Changing the number of nodes in the simulations does not change the features of the results noted.

Figure 1 shows a set of 4 plots that describe the effects that a non-polarized discussion has on the ability for nodes to initiate a content spread response, in comparison to the effect that a polarized discussion may have on that ability. Panel (a) is a heatmap where each cell is the accumulated number of ‘successful message propagations’ *s*_*n*_ (described in detail in the Supplemental Materials; the number of times a node *i* had an edge to another node *j* that subsequently produced content). As nodes produce and attempt to disseminate content the simulation records the successful transfer of content *s*_*n*_ between edge pairs (*i*, *j*) and presents that accumulation in the heatmap. This spread of information, by creating links, is counted in each entry and proportional to the color scheme in the legend. Nodes of lower id numbers began with lower *influence* scores also show lower counts on the ability to instigate message propagation in other nodes by the directed edges which are originally created according to a uniform Basal rate. Nodes with higher id values which began the simulation with a larger influence score, have a higher accumulated success in producing propagation responses. Panel (b) shows the equivalent heatmap of panel (a) but derived from the phase when there is the polarization introduced into the network. There is an interleaving (‘checker board’) effect due to the reduced probability a node has in having their content shared by anti-aligned members. The overall accumulations are also reduced in polarized phase as a result of situations where a node receives messages from both sides of the disagreement (‘mixed messages’ or ‘intimidation’ if the anti-aligned content is of high influence). Panel c) shows the ratio of the successful message propagations produced between the top half of the influential node ids (*n* ∈ {21, 40}) against bottom half of the node ids (*n* ∈ {1, 40}). During the course of the simulation where the node influence values can change based upon the success derived from message propagations driving up their perceived klout (Eq. 2). At the point where there is a dashed vertical line is the time when the polarization phase is introduced into the message exchange simulation and a change in the ratio of trend for the ratio of message successes can be seen from a decreasing trend to and increasing one. This represents that the initial nodes with higher node ids had a decreasing relative rate before the introduction of polarization, which then is regained due to the polarization. Panel d) shows the relative number of responses produced between each node id and node 1 for the non-polarized and the polarized phases. The key point here is that after introduction of polarization, the ratio of the successful messages that high ranked node ids are able to propagate to node 1 relative to that of low ranked nodes, increases, with respect to the same ratio before polarization.

The key features of the results from Fig. 1 are that during the simulation in panel (c) a decrease in the relative rates of message propagation success can be seen as a trend for the pre-polarization phase. This means that over time, nodes which start off with smaller influence values, can reduce their effective distance. This can be due to the mechanics of the stochastic message propagation algorithm allowing nodes with low influence to send a message to another node during the same time iteration that coincides with the reception of a message from a highly ranked node id. This effect represents a type of ‘piggy-backing’ that can occur with content repackaging or replication (retweeting maintaining or removing originator credit ‘shadow re-tweet’). As the number of message propagations increases over time the initial difference becomes less consequential and therefore within this framework we can postulate that non-polarized discussions produce a more uniform discussion between all members. This changes during the polarization phase in which nodes that begin with a relatively higher ability to disseminate content succeed in regaining their original power brokerage over spreading content. This can be attributed to the effect that contributions from different sides of the disagreement will cancel out their influence score contributions; as a form of intimidation or uncertainty that neutralizes the probability to fire (Eq. 4). What can be seen in panel (d) is that node 1, which has the lowest influence value to begin with has a greater barrier in which to disseminate content to all nodes in post-polarization phase. The result is that this node has little potential to produce a visible response in other nodes and fewer chances to amplify their content messages. This reinstantiation of the initial state of the influence effect, can therefore be used as a facility for the nodes which lost their influential klout, through a dilution of necessity in their dominance of the network, to regain influence by instigating or supporting the polarized disagreement. This can then re-establish their content as a larger proportion of the dissemination.

Figure 2 shows an equivalent plot as produced in Fig. 1(c) in the context of 3 phases of the discussion dynamics. The first two phases are the same as Fig. 1 where in the first phase there is communication between the nodes without any disagreement, and the second phase is where the polarization event occurs and the perceived influence is directly counter productive for disseminating content to the users on other side of the argument. In the third phase the innoculation strategy 1 (Subsection 4.1) is introduced in order to reduce the effects of the polarization. The effect of polarization is particularly the accumulation of a subset of the network that has an increased success in their ability to propagate content through intermediaries. This inoculation strategy, simply referred to as ‘inoculation strategy 1’ is aimed at re-introducing the trend towards a more uniform influence ratio between the upper and lower ends of the ranked influencers. The results of applying the inoculation strategy 1, show that the inoculation is able to reintroduce a uniform influence across the nodes as was the trend prior to polarization giving more chances for nodes on the lower end of the initial influence score distribution to disseminate content. As a result there is a greater defence against isolated interests of a small node subset.

The results from applying Inoculation strategy 1, described in Subsection 4.1 and defined in Eq. 5, shows a reversal of the influence disparity produced from the polarization period prior to it. Although the nodes which are ranked in the upper half of influence range still engage in the polarized content exchange, the lower ranked nodes can unilaterally decrease the disparity. This does not require any specific content subcategorization other than the apparent neutrality perceived by the messages sent to other nodes.

Figure 3 shows the results of applying the inoculation strategy 2 after a polarization event. The simulation is analogous to that of Fig. 2 where before the first vertical dashed line is a phase of non-polarized communication, and polarized exchanges respectively between the 2 verticle lines. The third phase of communication is when this second inoculation strategy (Subsection 4.2) is applied. This inoculation strategy 2 aims to place communicators in tiers where their communication is performed only between other users of similar influence values. As with the previous figure the trend of the accumulated bias for messages to be propagated when originating from high end node ids is reversed onto a direction supporting a more uniform message dissemination ratio. We can see that the rate of the decrease in the skew of the distribution is larger in this approach. Although these results demonstrate a superiority of the rate of improvement, this does require a participation from the platform administration, in comparison to a campaign towards those with little incentive to continue a polarization movement as their return is negative in terms of influence. The key component of inoculation strategy 2 is that there is a tiered separation of influencer groups and that it does not require an isolation of users due to content clustering. That the separation is geared toward restricting content from users which may produce large intimidating labeled content due to high influence rankings rather than any other aspect. It can be expected that users of a lower influence score may not find their voice to be substantial in participating in a discussion thread led by a user with more followers and a longer history of finding an audience of supporters, when their opinions are in disagreement.

The results of Figs. 2 and 3 show that two inoculation strategies that can appear seemingly different allow for a reinstantiation of the trend towards uniform content dissemination. Given that domination of content dissemination of today’s online social media landscape is a potentially valuable asset in certain hands, it may be misused. The results of simulations of the polarized content disseminations show how influencers losing control of the content dissemination in a network can adapt or instantiate polarization as a means to regain the relative klout derived in previous phases. Even if the concept is not known to those influencers, the changes to their effective dissemination rates will be monitored attentively and an effort to change strategies can be anticipated. In such a situation a more a ‘aggressive’ approach from the highly ranked influencers may be adopted in order to prevent their followers from disseminating content from other influencers. The approach of disseminating polarized content to maintain influence can be expected to be explored and tested. Creating negative outlooks on the content of the influencer competition, and their ideas can trickle down to the supporters which can instigate a simple way to divide groups.

## Discussion

The developments in the area of online social networks have brought a hallmark in the way in which humans can communicate and there are many ways in which their behaviors can change as a results of rapid cheap communication across the globe with high bandwith audio visual content. Maintaining the positive components of the interactions upon these digital platforms should be a high priority given the large populations that interact through them in most developed and developing nations. Since users can use the platforms in an effort to establish their influence due to the re-sharing features, a small group may dominate the discourse, and allowing more users to have an equal contribution is a key component to an open dialogue that should be supported. Therefore, in the situations where a uniform distribution is at threat, strategies to mitigate the effects have been explored in this work. The model explores how a set of established dynamics for the exchange of messages in online social networks together with a plausible incentive exchange for content dissemination can provide a reward structure for influencers to introduce polarization. The simulations show how polarization can allow for high ranked influencers to maintain their social dominance and gain a relative ranking from aligned followers which as a by product reduces the ability of others to disseminate their own content. Within these simulations a set of approaches are then explored, in which these effects are reduced and reversed effectively.

There are 2 inoculation strategies proposed that do not depend upon messaged content being visible for inspection or other specifics for the preservation of the privacy of the users. The key feature in both strategies is that the ‘intimidation’ factor represented by high profile polarized content is bypassed. It is bypassed with inoculation strategy 1 by non-participation from lower end nodes that have little to gain in terms of klout, which can be induced through recommendation campaigns, or from the inoculation strategy 2 which that places users into tiers of similar influence scores regardless of the polarized sides. These two inoculation strategies are shown to be effective applications of non-invasive and privacy preserving approaches that offer an avenue to reducing polarization. During non-polarized discussions, or with the inoculation strategies in effect, lower end influencing users can ‘piggy-back’ and during the ‘polarization’ intimidation plays a large role. The polarization dynamics are not directly affected or addressed as this may introduce complications that are unforeseen and induce possible effects of ‘back-fire’ during aggitated exchanges^22^.

The dynamics for the production of this effect can be used to identify reductions in message dissemination capabilities for the majority of social network members and to connect this with discussion leaders who utilize polarized content to increase their relative influence. Future work entails considering a similar framework but for the well known ‘celebrity scandals’ and how scandals can acutally elevate the popularity or influence of an ‘iconic’ member of society. This may require a definition and manipulation for what is considered to be a hazard towards a person’s identity. An extension would also possibly benefit from dealing with users that can have more or less numbers of links to create direct messages to users.

## Methodology

Information exchanges which have temporal dependencies rely upon a series of knock on effects. It is assumed that these events of a response are generated through a local decision criteria and are not aware of the mean field effect of the identical content impact elsewhere in the network. Although the individual considerations regarding a node’s discretion on whether to propogate a message it receives or not is a complex process. The macro behavior of the knock on effect is the main focus. The parameterization for these decisions are represented as a parameter of each node which is its *influence value* ranked among the other nodes; ${l}_{n}:0 < {l}_{1}\le {l}_{2}\le \ldots \le {l}_{N}$. Although this may seem simplistic, it does represent a ranked list for characteristics of popularity, influence, and other effects such as authority when this is directly linked to the response of a content dissemination event.

It is assumed there is a Basal rate for which nodes generate new content, *b*, as a probability which is uniform across the network and time. These events produce *c*_*b*_ events uniformly distributed to all the other nodes. Each event is realized as the production of a directed edge and *c*_*b*_ is the number of those directed edges. Upon being a destination node for an edge, the probability response function is defined for a node at a time point *k* as:

1 $${r}_{n}^{[k]}:\,=\frac{{\sum }_{i=1}^{N}\,{l}_{i}{({A}^{[k]})}_{(i,n)}}{1+{l}_{N}{\sum }_{i=1}^{N}\,{({A}^{[k]})}_{(i,n)}}\mathrm{}.$$

Each time a node will respond, a successful spread of (assumed related content) occurs (${s}_{n}^{[k]}$), creating *c*_*r*_ links uniformly chosen across the whole network. Implied in this response effect is a temporal dependency of content association. It is connected to the motivation for network members to obtain rewards to sharing important information with other members in a timely fashion.

We add to the model a mechanism of rewarding participation in stimulating a successful response in another node. This is done by having an increment in a node’s influence value. By increasing the value *l*_*n*_ this will result in more successful message responses in future directed edges produced. We impose that ${e}_{i,{\bf{j}}},i\notin {\bf{j}},|{\bf{j}}|={c}_{r}$ for unique edges to be produced and that they are not self directed. Any node that has an edge towards another node which produces a response in *k* + 1, can receive an increase (increment) of their influence value. The influence vector is not normalized across time steps since the denominator in Eq. 1, $1+{l}_{N}{\sum }_{i\mathrm{=1}}^{N}{({A}^{[k]})}_{in}$, provides for a relative measure, and social media platforms in general are not known to scale the analytics of popularity (friend number, retweet numbers etc) according to a network aggregate that is visible to the users.

A low ranking node in terms of perceived influence incurs the same increment upon their *l*_*n*_ value when inducing a response through a directed edge. In practice this may differ in how other nodes see incoming messages (content streams) either digitally or through traditional social interactions. The increment will be attributed due to the successful responses induced, which is uniformly attributed and denoted as ${s}_{n}^{[k]}$ with the update:

2 $${l}_{i^{\prime} }=({s}_{n}^{[k]}\times {({A}^{[k]})}_{in}+{l}_{i}).$$

This provides the motivation for the content exchange activity between users and effort to participate in the influence over other nodes in the network. As these increments will enable a user to have longer range of content exposure and recognition feedback.

It can be expected that over many iterations the initial max difference in node influence *max*(**l**) − *min*(**l**) would become less significant as increments are uniform across users. This difference can be assumed to remain constant over multiple simulations and if *max*(**l**) − *min*(**l**) = *diff* is considered to be approximately a constant this value for large *k* becomes insignificant in comparison to the minimum influence value of a future network state *min*(*l*_*i*_) $\gg$ *diff*. With a uniform addition of influence that is accumulated due to ‘piggy-backing’, or ‘mirroring’, from content and dark retweeting (removing original authorship labels) the minimization of the initial starting points is anticipated. This reflects the idea of a hypothetical ‘ergodic’ uniform state destination for a network of users with full visibility over enough time.

To represent a polarization phase; each node is put into one of two different groups, the *odds* and the *evens*. There should be an approximately equal amount of total influence for the nodes on each side. This break in homogeneity affects the response function probabilities by changing the perceived influence from each node from a different group. Taking the case where $mod(n,\,\mathrm{2)}=0$, *n* is on the ‘even’ labelled group, the polarized influence contributions for its response function becomes:

3 $${{\bf{l}}}_{even}=\{\begin{array}{ll}{l}_{i} & i\,{\rm{is}}\,{\rm{even}}\\ {l}_{i}\times (\,-\,\mathrm{1)} & i\,{\rm{is}}\,{\rm{odd}}\mathrm{}.\end{array}$$

The respective columns in the adjacency matrix used by each calculation of the response function becomes:

4 $${r}_{n\in even}^{[k]}:\,=\frac{{\sum }_{i=1}^{N}\,({({A}^{[k]})}_{(i,n)}\cdot {{\bf{l}}}_{even})}{1+{l}_{N}\,{\sum }_{i=1}^{N}\,{({A}^{[k]})}_{(i,n)}}.$$

With the swapping of the labels needed for the odd case. Having this positive in-group weighting and negative out-group weighting while maintaining the ability for nodes to send messages through transient links is explored in the Results section.

### Inoculation Strategy 1

The methodological extension provides for a method in which members of the message exchange network can increase their ability to disseminate content through other members. This is represented by their influence values, *l*_*n*_, and the state of polarization will inevitably change the distribution across the nodes. An approach which seeks to revert the relative influence values accumulated to that which produces more uniform response distribution is outlined. Drawing from approaches such as^20^ that develops a framework to reduce the effect of the ‘echo chambers’ it can be seen that link recommendation that focuses upon diverting antagonistic participation between sides based upon the content is potentially an effective strategy. Looking at the experimental results of ^37^ which examines a case study of exposure to opposing sides of an argument, a methodology which follows these directions is proposed which is mindful of practical constraints.

Given that Eq. 4 takes into account only the relative *l*_*n*_ values for the probability of firing due to external influence, the inoculation strategy alters the allocation of the influence values in the polarization stages from Eq. 3. The alteration is to ‘neutralize’ the content arriving from nodes in the bottom half of the network’s influence rank:

5 $${{\bf{l}}}_{even}=\{\begin{array}{ll}{l}_{i} & i\,{\rm{is}}\,{\rm{even}}\vee {{r}}_{{{l}}_{{\rm{i}}}} < {\lfloor }^{{N}}{/}_{2}\rfloor \\ {l}_{i}\times (\,-\,\mathrm{1)} & i\,{\rm{is}}\,{\rm{odd}}\wedge {{r}}_{{{l}}_{{\rm{i}}}} > {\lfloor }^{{N}}{/}_{2}\rfloor \end{array}$$

where ${r}_{{l}_{i}}$ is the rank of *l*_*i*_ amongst other nodes. This is an alteration on the Eq. 3 and models the effect that only the nodes regarded with the upper half of the influence ranks participate in exchanging content which is considered polarizing. Messages between the polarized sides that are not in the top half of the influencer rank can therefore have equal treatment in content value for propagation.

A practical application of this strategy in an actual social networking platform requires a information campaign of promoted content in order to bring awareness about the lack of return on a continuing disagreement. Given that these users may not participate proportionally in a ‘heated exchange’ of content, this may catch on and allow the previous state to return. This does rely on a funding for a campaign, or even that there is not rooted antagonisms between sides. Therefor the inoculation strategy 2 (Subsection 4.2) provides an approach which does not rely upon user understandings plus good will, but it does depend upon a platform’s policy in participating in an effort to reduce polarization.

### Inoculation Strategy 2

In contrast to the strategy proposed for the inoculation strategy 1 (Subsection 4.1), the strategy here addresses the issue that participants may not appreciate the information campaign aimed at reducing the effects of polarization upon their communication successes. While still maintaining the requirement for content privacy to be respected this approach does require participation by the platform administrators/developers. The concept behind the strategy is establishing a set of *tiers* based upon the influence values. That users are exposed to messages within those tiers only and does not separate according to polarization participation either. This is aimed directly at reducing the ‘intimidation’ factor without eliminating the concept of influence competition which drives much of the exchanges. By segmenting the influence distribution into tiers, users have a lower barrier of entry in the conversation by having smaller differences in the perceived influence values of the content based upon a user’s historical track record of producing disseminations.

Here we modify the response equation of Eq. 1 to represent the dissemination probability upon receiving content from other network members within their predefined tier. The tiers are not assumed to be calculated in an ‘online’ fashion where there is a continuous updating mechanism but that there are periods in which this can be reassessed. The locality for the simulations conducted here are based upon segmenting the network into 5 tiers (inspired by the 80–20 rule) where $\tau =\frac{N}{5}$ represents the size of the tier. The response equation is defined as:

6 $${r}_{n\in even,m={\lfloor }^{n}{/}_{\tau }\rfloor }^{[k]}\,:\,=\tfrac{{\sum }_{i=\tau \cdot m+1}^{N^{\prime} =min(N,\tau (m+\mathrm{1))}}\,{({A}^{[k]})}_{(i,n)}\cdot {{\bf{l}}}_{even}}{1+{l}_{min(N,\tau (m+\mathrm{1))}}\,{\sum }_{i=\tau \cdot m+1}^{N^{\prime} =min(N,\tau (m+\mathrm{1))}}\,{({A}^{[k]})}_{(i,n)}}\mathrm{}.$$

The introduction of the term, $m=\lfloor n/\tau \rfloor$, represents the tier number that the user is a member of. A possible disagreement from users who will experience this platform change can be in that it is a restriction upon the freedom of uniform association. An alternative perspective which the authors only speculate at how that can be addressed is if it can be made into a method of gamification^38,39^ in which levels provide an ability to strive towards another tier.

## Supplementary information

Supplementary information Polarization in social media assists influencers to become more influential: analysis and two inoculation strategies
