# Supplementary material for: Polarization in social media assists influencers to become more influential: analysis and two inoculation strategies
Source: Sci Rep. 2019 Dec 9;9:18592. doi: 10.1038/s41598-019-55178-8 (PMC6901574; doi:10.1038/s41598-019-55178-8)
Supplement: Supplementary file 1 — Supplementary information Polarization in social media assists influencers to become more influential: analysis and two inoculation strategies [file 41598_2019_55178_MOESM1_ESM.pdf]

Polarization in social media assists influencers to become more influential: analysis and two inoculation strategies

Ivan Garibay*[1,*], Alexander V. Mantzaris[2], Amirarsalan Rajabi[1], and Cameron E. Taylor[3]

[1]University of Central Florida, Complex Adaptive Systems Laboratory (CASL), Orlando, 32816, USA
[2]University of Central Florida, Department of Statistics and Data Science, Orlando, 32816, USA
[3]University of Central Florida, Department of Computer Science, Orlando, 32816, USA
[*]igaribay@ucf.edu

ABSTRACT

The material supplied here covers the details for the dynamics of the simulations in which messages are propagated between users in the online social network. A key feature is how a score is attributed to each user based upon the ability to spread their message content by having other nodes amplify their content.

Supplemental Material

When simulating an exchange of messages between users in the synthetic social network, a 'score' for the total number of successful messages a users manages to instigate in other users is an indicator of influence according to the model paradigm proposed. Using a heatmap, the scores of successful message propagations can be cross compared between the different id numbers. The heatmap cell score values are calculated using;

$$score_{i,j} = \sum_{k=1}^{T} s_{i \to j}^{[k]}. \tag{1}$$

Here s is the item indexed array for the successful responses $r_{i \to j}^{[k]}$ at time point k.

The simulations are also monitored by examining a bar chart for the relative successful responses generated by the network members in comparison to a single user. The heights for these bars are measured by the response influence pre and post polarization by the following:

$$score_{i,T'} = \frac{\sum_{k=1}^{T'} s_i^{[k]}}{T'}. \tag{2}$$

The ratio of the node triggering capability with another node is:

$$\frac{score_{i',T'}}{score_{i,T'}}. \tag{3}$$

Here we choose i to be node id 1 for the results displayed.

To measure the running average of one group's ability to initiate message spread in comparison to other remaining group a sum over the $s_n^{[k]}$ values is produced. Given a subset of nodes N_{top} from the total number of nodes N the rest of the nodes in the network are $N_{bottom} = N - N_{top}$. The average number of node message spreading is measured from the first time point to another time point at intervals of 1K iterations with the final time point being T and itermediate time points at T' (for the top nodes):

$$score_{top} = \frac{\sum_{k=1}^{T'} \sum_{n=N_{top}}^{N} s_n^{[k]}}{T'}. \tag{4}$$

For the ratio of the top to bottom:

$$\frac{score_{top}}{score_{bottom}} = \frac{\sum_{k=1}^{T'} \sum_{n=1}^{N_{top}} s_n^{[k]}}{\sum_{k=1}^{T'} \sum_{n=1}^{N_{bottom}} s_n^{[k]}}. \tag{5}$$

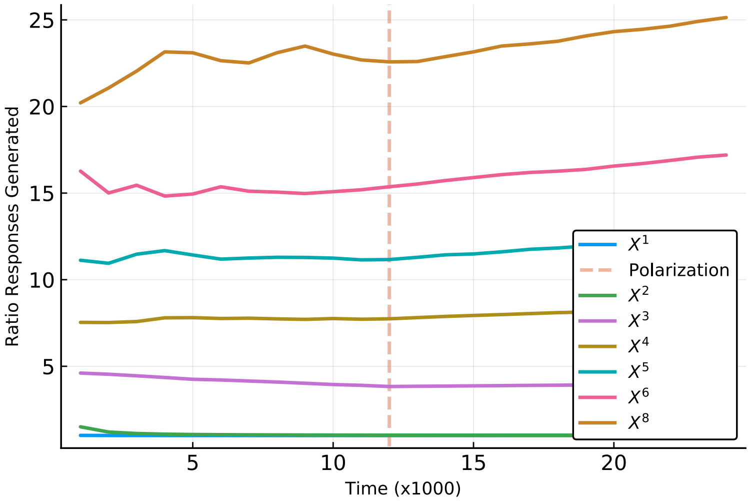

Figure 1. An exploration of the initial influence score distributions and the changes in the initial response ratios and simulation traces.

In the simulations presented in the Results sections n_{top} is chosen to be $N/2$ (20) nodes. Figure 1, shows an exploration of different intial influence score distributions applied to the nodes. The vertical axis is the difference in the responses generated between halves of the nodes as demonstrated in the Results section. The skew of the distribution produces more initial disparity and this does have consequences for the simulation trajectory on if that disparity is enough to drive a further increase or not.
